# What a State: Why the U.S. is Still Bad for Your Health (Policy)

**DOI:** 10.1002/hpm.70009

**Published:** 2025-07-09

**Authors:** Calum Paton

**Affiliations:** ^1^ Emeritus Professor of Public Policy School of Social Political and Global Studies Keele University Keele UK

**Keywords:** conservative bias, obamacare, political institutions v ideology, the U.S. state, trump

## Abstract

The second Trump administration's centrepiece legislation, the modestly‐named Big Beautiful Bill, passed by the House of Representatives and going through the Senate at time of writing, offers an opportunity to reflect upon how the U.S. state affects health policy and the prospects for equitable access to affordable healthcare. Is the U.S. still an outlier (by comparison with Europe and much of the world), in that its many of its citizens are either uncovered, poorly covered or tenuously and only temporarily covered by health insurance? The answer is yes. And the chipping away at Obamacare and Medicaid by Trump 2.0 (learning from his failure to repeal Obamacare in 2017) as part of the Big Beautiful Bill, shows us that it is easier for the Right to dismantle progressive social legislation than it is for the Liberal‐Left to assemble it. To understand why, and to revisit why the U.S. polity struggles to enact progressive healthcare reform, we have to understand the effect of the U.S. state (i.e. political structure) upon public policy. This article revisits the nature of that state, to depict the underlying causes of ‘American exceptionalism’ which are partly ideological but also more significantly institutional than often realised.

## Summary

- Shows how the U.S. state biases health policy in a conservative direction.
- Trump's Big Beautiful Bill will further weaken ‘Obamacare’ (the Affordable Care Act) by weakening Medicaid significantly.
- Institutions are as much as a cause of American exceptionalism as ideology; the U.S. state's effect upon health policy is perhaps the key example.
- The main international lesson is that political structure can facilitate or retard effective health policy, and facilitate or retard conservative attempts to weaken equitable national healthcare systems. The U.S. state is quite exceptional by comparison with Europe but, for countries forging new constitutions or changing their structures, the problems encountered in the U.S. should offer a warning.

## 1 Introduction

It has been claimed that the conventional 'paradigms' of American politics are all lacking something, and that that ‘something’ is the effect of U.S. political institutions in creating a 'mobilisation of bias' against coherent social planning [1]. Health policy is the classic case [2].

The conventional paradigms include cyclical theories of U.S. politics ‐ simple ones such as Schlesinger's [3], which posited a pendulum swinging between liberalism (in the modern U.S. sense) and conservatism; and complex ones such as Huntington's [4, 5], which posited a four‐stage cycle embracing *complacency, idealism, hypocrisy and cynicism.* Huntington, for his part, had suggested that the motivation for liberal reform was to close the *gap* between ideals and institutions, producing periods of ‘creedal passion’ when reformers sought to ensure that institutions lived up to America's loftier ideals.

Huntington therefore accepted the impetus to liberal reform as important in U.S. politics, but incorporated it into a more complex framework, as the next sentences explain. When reformers' goals were achieved yet reformers kept complaining of the *gap* characterised hypocrisy in Huntington's schema. He gave the example of the radical movements in the late 1960s which took little account of the real achievements of President Johnson's ‘Great Society’ legislation ‐ which included Medicare and Medicaid. One could add the ‘woke’ agenda of today, which has been in part responsible for the poor performance of the Democratic party at the Presidential level and the rise and re‐rise of Trump. In Huntington's schema, in other words, hypocrisy led to cynicism.

Huntington's theory contained much insight, but it did not always map onto the actual trajectory of American political history. For example, the ‘cynicism’ which followed the Great Society, perhaps embodied in the Presidency of Richard Nixon, who was elected in 1968, did not end the cycle and lead back to complacency, as his schema would suggest. Instead, an idealist, Jimmy Carter, was elected in 1976, two years after Nixon's resignation. More importantly for our purposes, Huntington's schema did not account for how policy itself ‐ and its nature ‐ affects the American political cycle.

It did not account for *either* why U.S. social planning is an outlier in the developed world, in terms of its incomplete and hesitant nature even in periods of liberal reform, *or* how policy outcomes over time, themselves affected by the nature of the U.S. state, created an ongoing bias against coherent social planning for example reform of the health‐care system. The U.S state is dysfunctional for progressive social planning ‐ as opposed to progressive single‐issue regulation, which it can achieve quite effectively in periods of liberal reform (liberal in the modern, American sense, of course.)

Paton's original argument was a corrective to the dominant view that 'ideology' and 'American exceptionalism' were responsible for the distinctiveness of U.S. policy when the latter was viewed in a comparative context. That is not to deny that the public policy literature was nuanced about the determinants of policy [6] and also that state theorists such as Skocpol [7] had a different perspective from the classic depiction of the American polity. But despite all, the Hartzian [8] view of the U.S. as uniquely liberal, and ‘exceptional’ in that sense, remained dominant.

## 2 Weak State, Weaker Policy

Paton [1, 2] had argued that traditional accounts of the nature of the U.S. state were inadequate to explain its effect upon public policy, particularly policy which sought to apply social planning in combining equity and efficiency in social reform (e.g. in health‐care reform.) In terms of the ‘ideas v. institutions’ debate [6], he argued that a ‘mobilisation of bias’ (taking the phrase of Schattschneider [9] in a different sense) engendered by the operation of the U.S. state's political institutions ‐ beyond that implied by the formal, constitutionalist reading of those political institutions ‐ was harmful to coherent reform.

Moreover, while more ingenious cyclical theories of U.S. politics [4, 5] were more insightful than simpler cyclical accounts of U.S. politics [3], they had two flaws. Firstly, they did not account for how the nature of policy created a kind of path‐dependency which vitiated coherent reform. Secondly, Huntington [5] argued that periods of what he called ‘creedal passion’ (which we might call an impetus to liberal‐left reform) weakened the state.

Paton argued [1] that Huntington got things the wrong way around: in fact it is the weak U.S. state, and the ‘biases’ to which it tends, which explains why progressives often found the federal state inadequate for their purposes. True, progressives ‐ and populists of the Left ‐ have (in line with American exceptionalism) been suspicious of the national (i.e. federal) state for a variety of reasons, in earlier periods of American history. And even today, radicals and left‐liberals often seem hostile to the federal state. The populist Right, of course, was *and* is hostile to an active federal state in domestic policy. Musk with his chainsaw is only the latest symbol of this.

However, as Morone has argued [10], the reforming liberal‐left needs the federal state ‐ especially in the modern age, one might argue, when reform has to tackle national and international economic forces. Reform today occurs within the context of an industrial and indeed post‐industrial economy (not more local agrarian reform et al.) as in earlier progressive eras. The local state, on the one hand, and protest movements, on the other, are powerless to tackle the roots of economic power in the modern environment.

There has long been in the US a suspicion of ‘bureaucracy’ and a perceived need to keep it under political and social control in a manner that is (e.g.) quite foreign to those in the UK who see a politically‐neutral, stable and even ‘autonomous’ civils service as a good rather than a bad. This can arguably be traced right back to the Constitution. Yet the fear of bureaucracy has made those bureaucracies which *are* necessary to administer social programmes more partial, temporary, insecure and ‘captured’, which ironically makes them less effective ‐ and, doubly ironically, less legitimate. Thus it is easier for Right populism to attack them and even sweep them away, a point which may well have resonance in the era of Trump.

Additionally, progressives have in American history sometimes seen the federal state as elitist ‐ perhaps fixating on the Hamiltonian view of the Senate as the chamber for the elite as opposed to the more representational House. But it is the difficulty in passing progressive legislation given the separation of powers which is the main problem today. And not just the separation of powers, but the veto‐points and access points for well‐funded interests *within* each legislative chamber and within the porous executive, not to mention the politicised legal system of the US including the Supreme Court at its apex.

In the UK we have seen the negative side of majoritarian governments which are unchecked by ‘checks and balances’. But the other side of the coin is, in the US, the lack of a sovereign parliament based on a sovereign people at the federal level. Sure, in theory, those checks and balances which hamper progressive reform may also retard reactionary attempts to sweep away those progressive achievements which *have* been possible. But knocking down a building piece by piece, incrementally, is possible; assembling a tower piece by piece *using different materials at each stage* may not work. Removing progressive 'bureaucracies' at a stroke, in exceptional periods of ‘crisis’, is easier than creating coherent reform with capacity for implementation (i.e. a coherent bureaucracy) at a stroke.

This is not to deny that there have been exceptional periods of progressive reform, such as the New Deal and Lyndon Johnson's ‘Great Society’. Even the former was distributional rather than redistributional, however, and an exercise in ‘Keynesian’ demand stimulation rather than social planning. Even the Great Society, for its part, despite the valuable achievements of (e.g.) Medicare and Medicaid, was characterised by significant compromise (e.g. Medicare and Medicaid instead of national health insurance.) And now, after the modest achievements of Obamacare, which built on the Medicaid programme we see it being gradually undermined in both financial and structural ways.

## 3 Weak Reform and Health Policy

Paton's thesis was also a corrective to arguments such as Huntington's that periods of reform from below inevitably weakened the state. Rather, it was the other way around: a weak state prevented reform from harnessing the power of state to achieve its objectives. Additionally, in the case of health policy in particular, timid and weak liberal reform failed to combine equity and efficiency, as it had to genuflect too much to the pre‐existing healthcare structure which was both expensive and fragmented, built in a piecemeal manner around sectional private interests. As a result, liberal reform disappointed in its outcomes.

The health planning legislation of the 1970s sought to rationalise the system in order to improve its efficiency as a precursor (in the minds of many of the liberal reformers who sponsored it) to enacting National Health Insurance on an affordable basis. But it did not achieve much, while nevertheless being portrayed as cumbersome by conservatives ‐ and, in hindsight, as trivial by many of those who had originally held out hope for it. This fed a conservative backlash as regards healthcare reform.

Thus there was a cycle from liberalism to conservatism, or from idealism to cynicism ‐ but not for the reason Huntington suggested, which was that liberal reform went too far and overreached itself. Almost the opposite, in fact: in compromising out of necessity with the status quo (given that that was the only way to pass legislation, given the *modus operandi* of the U.S. state), the 1970s healthcare planning reforms were too weak to achieve their objectives. As a result, extending access via National Health Insurance would indeed have been expensive. But this would not have been because it is intrinsically so; it would have been because the profligacy and waste in the predominantly private healthcare system had not been tackled as the proponents of the 1970s planning reforms had hoped.

This is arguably one reason why President Carter did not risk nailing his colours to the mast of National Health Insurance (NHI.). His healthcare bills were much more incremental, which disappointed Congress's leading proponent of NHI, Senator Edward Kennedy, who then ran in the Democratic primaries against Carter in 1980. He lost, but it further weakened an already‐weak Carter, who then lost the Presidential election to Ronald Reagan, who served from 1981 to 1989.

The rest is history. Through the 1980s, the healthcare reform debate was dominated by those who believed in market competition rather than planning. This philosophy underpinned, for example, the failed Clinton healthcare reforms, and even the timid Affordable Care Act (‘Obamacare’) of 2010.

This is not to deny that there were improvements to access. The Emergency Medical Treatment and Active Labour Act (EMTALA), passed in 1986 and implemented in 1988, ensured that individuals could receive emergency treatment regardless of their ability to pay. (This of course created a perverse incentive, in the absence of national health insurance: it created an incentive to get sicker before seeking care!) Another was during the George W. Bush presidency, via the Medicare Prescription Drug, Improvement, and Modernisation Act of 2003 (MMA), also known as the Medicare Modernisation Act. This extended Medicare to provide an outpatient prescription drug benefit, Medicare ‘Part D.’ But other clauses reduced the reimbursement available under Medicare more generally. Moreover, policies such as tax allowances for Health Savings Accounts, were minor when posed against the challenges of extending access, and arguably not a contribution to a*ffordable* healthcare.

Politics is of course about people, not just political structure. Exceptional circumstances produce exceptional opportunities. Beleaguered by the Watergate scandal, President Nixon sought to divert attention by doing the unexpected: he reached out surreptitiously to his political enemy, Senator Edward Kennedy, and offered a joint initiative to enact NHI. In his memoirs, Kennedy expresses regret that he did not swallow his contempt and mistrust of Nixon (who in turn loathed the Kennedys; he had of course narrowly lost the 1960 Presidential election to Ted's elder brother John) and agree. But such would have been exceptional, not normal, politics.

## 4 New Society, Old State, Weak Policy—Still True Today?

Huntington had earlier argued [11] that the US had a feudal‐type state in terms of political structure ‐ an ‘old state’ ‐ which sat atop a ‘new society’. That is, the US was *founded* as a new, or modern, society, and did not need to modernise using the centralised powers of a ‘new state’ in order to develop from an ‘old society’, as did Europe. Paton suggested that, when a ‘new state’ was required (e.g. in health policy), it was absent.

We may ask: how true is this characterisation today? And does the US *need* a more centralised federal state today? Do Trump's efforts in this direction in order to enact his agenda ‐ *l'etat, c'est moi* ‐ ironically suggest a blueprint for the Left?

It can be argued that, in the past, the difficulty which the US state (irrespective of which party controls all or part of it) has in redistribution as opposed to regulation and distribution [12] could be covered up and compensated by an *alternative* that is the US's ability to use its hegemonic position in the world economy (see below) to provide cheap goods and services even to citizens of modest means.

Trump's attempts to use tariffs to protect or restore American industrial jobs may also be an *alternative* to redistribution. After all, America's large trade surpluses from services, coupled with deficits in industrial goods, are simply the consequence of trade specialisation. Thus protecting the domestic losers (industrial workers and their communities) and helping them move to new forms of employment would normally be the responsibility of a national government. But the US state makes the progressive fiscal policy required to do this very difficult to achieve ‐ for Democrats and Republicans alike.

Trump is in effect saying, *we won't give you national health insurance, nor even retain Obamacare, but we will give you your jobs back.* Of course this is duplicitous: the Big Beautiful Bill redistributes spectacularly from the poor to the rich through the nature of its tax cuts and also through its attack on social legislation and spending. While some of Trump's electoral base may benefit from the tax‐spend reorientation, swathes of it will not. Polls suggest that two‐thirds of Americans support the preservation of Obamacare. And minor tax cuts for the less‐well‐off in work will only lead to a net benefit for some of these that is those who already have some form of employer‐provided or privately‐purchased health insurance. Moreover such insurance is precarious.

It can be argued admittedly that America's position as an outlier, even today, is not structural but simply the consequence of the dominant American ideology, augmented these days with the fact that the ‘anti‐globalist’ Right is fundamentally a populist movement which wants to cut the taxes of the rich disproportionately yet use other means to attract voters from the left‐behind (or those who feel left‐behind) in society ‐ such as anti‐immigration, for sure, but also the restoration of jobs and prosperity for the left‐behind communities.

In practice, it is likely to be *both* ‐ ideology and institutions in a symbiotic relationship. Even at times of liberal reform, when individualist ideology is less of a barrier, legislation is fragmented and cumulatively expensive as a result of U.S. political institutions ‐ with healthcare the classic case. So disillusionment which has an institutional cause feeds a backsliding towards conservative ideology.

In most advanced capitalist societies, conservative retrenchment of welfare states continues apace. But in the U.S. the institutional settlement gives it a further boost: better for pivotal social strata such as the tenuously‐employed or the not‐quite‐poor to seek benefits individually or stratum‐by‐stratum rather than hitching their waggon to a coordinated universal welfare train which is unlikely ever to arrive at its destination.

## 5 Saved by Economic Hegemony?

The US's dominant position in the world economy has meant in the past that it can use hegemonic economic policy to paper over the consequences of fiscal incontinence (high spending and low taxes). This fiscal incontinence has been greatest in times of Right‐wing ascendancy ‐ first Reagan and now Trump. The US can only solve this policy incontinence, exacerbated and in part caused by the structure of its national (i.e. federal) state, by ‘exploiting’ the rest of the world. We saw this with the Reagan deficits financed by Kissinger's hegemonic deals with the Middle East (as well as Japan's trade surplus). We are seeing an *attempt* with Trump's tariffs. If the tariff policy fails, then the U.S. will be exposed as requiring to modernise its state. It would need to solve its *political* problems (caused by e.g. loss of manufacturing jobs) through internal domestic means of redistribution, assuming that Trump's voters eventually saw (if tariffs fail) that voting with their anti‐woke gut rather than their rational head was a blind alley.

In todays' global age (whatever type of globalisation we mean), political economy is admittedly more important than state structure. But even so, the U.S. state may be increasingly anachronistic when it comes to the policy required even for a hegemonic economic power to operate efficiently.

In the 1980s, Ronald Reagan practised ‘reverse Keynesianism’ ‐ high expenditure (albeit through vast expenditure on the military, combined with cuts in social programmes) and low taxation. This was arguably because the alternative ‐ cutting budgets to match the tax cuts ‐ was not only politically undesirable to much of Reagan's constituency (defence contractors et al.*) but also* politically impossible. Reagan's first Budget Director, Dave Stockman [13], talked of the ‘hogs really feeding’ in the early Reagan budgets. This in turn reflected ‘politics as usual’ in Congress. There was no coherent social planning. But there was pork‐barrelling and log‐rolling a‐plenty.

And ‘reverse Keynesianism’, moreover, is the opposite of the real thing in the most important respect ‐ it increases inequality and poverty. There is another aspect to creating a stark budget deficit by de‐taxing the rich: it increases interest rates (and chokes off private investment based on borrowing, as well as clobbering your mortgage.) The only way that ‘trickle‐down’ does not lead to punishingly high interest rates is if there is cheap money or ready investment from abroad, without interest rates having to be too high to attract it ‐ as in the US, benefiting from Saudi oil profits and Japanese government surpluses in the 1970s. But even the US's economic hegemony almost 50 years ago did not save it *wholly* from high interest rates from the likes of Reagan running huge budget deficits. Chairman of the Federal Reserve Paul Volcker controlled inflation in the early 1980s by a double‐dip recession which wiped out swathes of manufacturing industry and left the US as Trump found it 40 years later ‐ running a strong Balance of Payments surplus in services and a stark deficit in manufactured goods.

Ironically therefore, the prototype ‘make America great again’ President ‐ Ronald Reagan ‐ destroyed the industrial ‘greatness’ which his ‘MAGA’ successor's (Donald Trump's) tariffs are intended to restore, in a huge gamble which has shaken the neo‐liberal world economy. If it works, then once again the US will have papered over its problems at the expense of the rest of the world. But if it does not, then the US state will have to confront the fact that coordinating a coherent economy in times of economic difficulty is barely compatible with ‘politics as usual’ ‐ unless ‘politics as usual’ means cuts to social programmes on a significant scale. And for health policy, that would mean that, as regards cutting Medicaid and Obamacare, ‘you ain't seen nothin’ yet’, to quote another conservative President, Ronald Reagan.

## 6 So How Does the U.S.’s ‘Mobilisation of Bias’ Work?

Classic political institutional theory tells us that the US Constitution was designed to make it difficult to pass (domestic) legislation without a large degree of consensus. Hence the separation of powers, including a powerful Supreme Court, and the complex structure of the legislature, including inter alia a Senate giving excessive powers at the federal level to small states (in terms of population, in that all states have two Senators irrespective of size). The U.S. regularly requires a ‘concurrent majority’ to pass federal legislation.

Paton's argument went further [2]. The *dynamic* effect of institutional stickiness and stasis created a self‐reinforcing bias: when legislative ambition was thwarted (e.g. to pass National Health Insurance) and resulted in (at best) compromised half‐way measures, political ambition was reduced over time to augmenting these half‐way measures with further (what we might term) quarter‐way measures. This provided an *institutional* rather than *ideological* explanation for timidity as regards ‘European‐style’ health and welfare policy.

It would be foolish to deny the ideological verve in the U.S. against ‘socialised medicine’ and the like. But the argument is that ‐ even at times of liberal ascendancy ‐ the institutional ‘problem’ restricts achievement. Moreover it makes that reform which is possible less effective and more costly (lots of atomistic health programmes, e.g.): this then encourages the conservative backlash which argues that ‘liberal reform’ is both undesirable and unaffordable. *Institutional bias augments and reinforces ideological exceptionalism.*

Let us consider what may happen to Medicaid and Obamacare, and how it is easier to undermine it than (say) to erode the British NHS. Trump's Big Beautiful Bill, the legislative centrepiece of his second term. seeks to remove the clause in Obamacare which allows anyone with an income up to 133% of the federal poverty line to access Medicaid (the healthcare coverage programme for certain categories of the poor) as long as states play ball. It also seeks to make having a job a condition of getting Medicaid. This would have the perverse effect of some people only getting eligibility for Medicaid when they didn't need it. And for those still eligible (on low incomes; or doing public or charitable works if they can't find a job), the conditions to register ‐ and re‐register every 6 months ‐ are designed to weed people out of Medicaid through putting up administrative barriers.

Some of this may fall by the wayside. But the point stands. And in any case, at the time of writing, the Senate is increasing the cuts to Medicaid which the House has agreed. Currently states can tax hospitals and doctors, then spend the money in the health sector attracting more federal funding (Medicaid is a matching programme which attracts federal funds in proportion to the level of states' funding.) The Senate version of the Bill would outlaw that.

Had the USA had an NHS or an established National Health Insurance scheme with stable funding, this attrition would be less possible. Timid, piecemeal legislation, ‐ often requiring regular re‐authorisation and/or re‐appropriation of funds ‐ creates a path‐dependency away from stable reform. It also is easier to destroy. And it means that left‐liberals spend much of their time in a rearguard action, further militating away from more ambitious reform.

## 7 Trumpian Politics?

In an age when the international economy is an external constraint upon most countries' scope for autonomous action, it is only in countries with the largest economies that states have substantial autonomy, at least in economic matters, which then of course has a knock‐on effect upon fiscal policy and therefore social policy. Trump's second Presidency has already demonstrated that. The question here is: to what extent do Trumpian attempts to by‐pass ‘normal politics’ in the U.S. suggest a model for the liberal‐left in politics? By‐passing the usual checks and balances could be useful for the left, could it not? After all, Huntington's argument about the U.S. being a ‘new society with an old state’ was based on the premise that the U.S. did not require to modernise in the way that European societies did; it did not *need* a strong state.

We should however be careful. Trump's centralism is based upon single‐issue executive orders. These may be useful for removing regulations, or imposing tariffs (especially if Congress and the Supreme Court are supine.) But this mode of governance may not be suitable for making complex law, with implications throughout the federal system, of the sort which would be necessary to ‐ for example ‐ create a national health service or national health insurance system complete with adequately‐planned provision of health‐care facilities on a national basis. It is, to reiterate, easier to use the U.S. state to remove bureaucracy than to create it.

It can be argued that the US state is more amenable to direct capture by specific capitalist interests or ‘strata’, against the interests of the whole ‘capitalist class’ or at least of the whole economy. For example, the US ‘healthcare non‐system’ is difficult to reform, not least on account of the direct lobbying of the provider and insurance industries, which ‘capture’ key Congressmen. This means that the business sector as a whole may be disadvantaged ‐ to the extent that it has to pay for (expensive) insurance for key workers whereas in (e.g.) the UK, the state pays through taxation which affects individual medium‐sized businesses proportionately less.

As a counterpoint, today we see stronger and more ideological political parties in the U.S. ‐ both Democrat and Republican. But even so, below the waterline of ideology and culture wars, healthcare politics remains both decentralised and gridlocked. Campaign finance, not least the 2010 Supreme Court decision which blows a hole through attempts to control the spending of billions, means that candidates in primary elections for the Senate and House of Representatives are less likely to challenge vested interests with a stake in private healthcare and private health insurance. We saw the consequence of this when Obamacare was watered‐down in order to be passed, a vitiation which was necessary despite Democratic control of the Presidency *and* both chambers of Congress. And even if a national political party has enough influence to corral candidates for the Senate and House, it is likely to be because of the money of powerful interests operating at national level (think Elon Musk.)

## 8 Is it the U.S. State or is it the Capitalist State More Generally That is the Problem?

The argument that the U.S. state is simply the most prominent Western capitalist state, and that *that* explains its health and social policy nature, is easily debunked. Those who still cling to the perspectives of Marxist state theory try to explain policy in terms of a generic ‘capitalist state’ or generic ‘state in capitalist society’ underplay how different state structures affect policy significantly. There is in fact no such thing as *‘the* capitalist state.’ There is not even a generic type of ‘state in capitalist society’. What there are are different types of state in different types of society, capitalist and otherwise.

The UK and some other European ‘capitalist’ countries, such as Denmark, Sweden, Norway, Finland, Spain, Portugal and Greece, have national health services (NHSs). Other European countries, and many other countries around the world, have national health insurance systems (NHI). Some Marxists seek to argue that these are just different ways of organising health services which are functional for ‘capital’, the difference from the U.S. being that, in the U.S., the capitalist nature of health services is simply more transparent. But this is far too reductionist.

This is not to deny that some of these systems may indeed be functional for ‘capital’, as the Marxists call business. For example, an NHS which provides healthcare efficiently and maybe even cheaply to the population through general taxation may be less burdensome to the majority of private businesses than healthcare systems such as that of the U.S. (where business often faces high health insurance bills for its workers, especially if the main approach by liberal reformers is to regulate the private sector through approaches such as ‘play or pay’, rather than replace private insurance with a public health service.) But that does not mean that an NHS's *primary function* is to aid capital: Europe's NHSs are much, much *more* than the state's role in helping capital. They are expressions of both social solidarity and successful reform in the interests of working people.

In the U.S., it is of course true that private firms will generally insure their workers only if it makes sense economically to do so. That is, it is worth their while to invest in scarce skilled workers (whom it takes time and resources to recruit, train and develop) by offering them inter alia decent health insurance; healthcare may even be a recruitment tool. Plentiful unskilled workers, on the other hand (whom it is easy to replace) may simply go without insurance.

Therefore it *is* possible, at a stretch, to depict the U.S. healthcare system as ‘functional for capital’, to use Marxist terminology. But unlike in countries with national healthcare systems, this does not directly involve the state investing in firms' healthcare, unless one counts various tax‐incentives. In the U.S., state‐funded programmes such as Medicare and Medicaid are by contrast substantially for the elderly and the poor.

In any case, such a depiction is too functional*ist* in that it attributes a logic to the system which it does not have. What we in fact see in U.S. health policy is a weak state which *neither* coordinates sectional interests in the interests of capital overall *nor* achieves a national health system which promotes equity in an affordable manner.

Theorists who still cling to (what they call a) Marxist view of the ‘capitalist state’ have been forced to abandon the simplistic and discredited Marxist view of the state as the direct instrument of the capitalist class. Today's academic ‘Marxists’ tend to see the nature of the state as determined by prevailing ‘social relations’, by which they seem to mean the relative power of different social classes at different times and in different places. ‘Strategic selectivity’ developed as part of a so‐called ‘strategic‐relational approach’ [14] is the idea that state and other structures are not neutral but favour some actors, strategies and/or outcomes over others. This (if one can interpret what is often obscure and tortuous language) would seem to imply that the differences between healthcare systems such as (say) the UK's NHS and the U.S. system reflect different social and class relations. ‘Strategic selectivity’ operates in both, but has different results as there are different power relations at work in different polities.

The trouble is that the jargon around ‘strategic selectivity’ and the like is merely a new language designed, consciously or subconsciously, in a vain attempt to salvage some life from the corpse of Marxism. (We may note that Anglo‐American political science was quite capable of analysing ‘non‐decisions’ and biases without recourse to Marxism.) What is more, ‘strategic selectivity’ is itself a watered‐down version of another concept, ‘structural selectivity’, developed from earlier Marxists [15, 16]. At root, Marxism assumes (a) that there is a cohesive ‘exploited’ working class with an objective interest and (b) that if this interest is subverted or obscured, then the state must either be causing this or condoning it. Those who want to explain reality yet stick to Marxism therefore analyse the state's nature and the policy it produces with their conclusion already assumed.

But *neither (a) nor (b) is necessarily true.* To seek to analyse the biases within state decision‐making and policy‐making within the theoretical trajectory of (quasi‐) Marxism is unnecessary (and also likely to produce reams of barely‐readable text, as the conflicted author seeks to square umpteen circles!). Marxists see the state as a coordinating mechanism to ensure ‘capital accumulation’ under capitalism, given that individual capitalists/enterprises are too short‐sighted to do this, a perspective which goes all the way back to Engels.

The problem for Marxists is that the state can be *more* than this. It can also be less than this: in the U.S., the state is often incapable of being even the “the executive of the whole bourgeoisie” in the words of Marx and Engels. This is said in light‐hearted, hyperbolic fashion, but the kernel of truth remains nonetheless. Even the era of neo‐liberal global capitalism may be coming to an end, with Trump merely a straw in this wind. Thus the idea that neo‐liberalism is the final nail in the coffin of a progressive state anywhere in the world is, like the report of Mark Twain's death, an exaggeration!

To the Marxist, any state policy or decision which advances social equity or other progressive objectives has to be interpreted as atypical or unstable. (This is tied up with the Marxist view that progressive policy is, at best, ‘legitimation’ of capitalist economy [15].) My point is that progressive policy *is* more likely to be atypical and unstable in the U.S. than in other capitalist societies, but that this is explainable without recourse to any quasi‐Marxism.

In the U.S., the nature of its (comparatively) unusual state is responsible for the instability of progressive reform. Moreover the state's frequent inability to be an effective ‘coordinating’ mechanism to render progressive reform coherent and fiscally manageable is responsible for conservative reaction of the sort which Trump's Big Beautiful Bill embodies, notably in terms of its cuts to public healthcare. Moreover, the U.S. state is often unable even to coordinate a coherent policy for business (for ‘capital accumulation’, as Marxists would have it). To date, its global economic hegemony has rescued it, as discussed above. If this is threatened (as may happen either as a result of or despite Trump's mercantilist policies, as well as the rise of China et al.), then the state's dysfunctionality may loom larger.

Marxism would indeed seek to explain away societies' social accomplishments as aberrations, when they may well not be. Indeed quasi‐Marxism explains away the whole edifice of post‐war Keynesian welfare states as ‘Atlantic Fordism’, merely a temporary ‘regime’ which suited capitalism at the time in its nationally‐based incarnation but which has been superseded by globalisation which is also known to certain Marxists as the ‘Schumpeterian workfare post‐national regime’ [17] On this reading, Thatcher and Reagan were merely the midwives of capitalism giving birth to a new regime.

This makes a nice story, but its truth ought to be interrogated sceptically. Let me explain why, by comparing the U.S. and the U.K. For (in good comparative style by which we understand something by showing what it is not) we may grasp the nature of the U.S. state by contrasting it with the UK. Let us take the example of the UK in 1979, when the post‐war Keynesian consensus had allegedly broken down. The oil shocks of 1973 and 1979, exacerbated by deadlocked industrial relations (the failure of trades unions to accept wage restraint) allegedly led to the decreased viability of ‘social democracy’, ‘Keynesian demand management’ or ‘corporatism (call it what you will.) Marxists tend to argue that capitalism's declining viability meant that it ‘had to be’ reinvigorated through a new type of regime ‐ with Margaret Thatcher as its midwife in the UK and Ronald Reagan following a year later in the U.S.

But post‐war ‘social democracy’ was not *destined* to fail. The reason it did was that, at the time of its abandonment, it had hardly shown itself in a great light; the electorate (having seen both main political parties attempt to manage the economy through corporatist means with limited success) had lost patience. There was nothing inevitable about Thatcher or Thatcherism. And even in the 1980s, Labour was free to offer a radical alternative, as it did (very imperfectly) in 1983. But the British electorate did not see this as viable, probably quite rightly, and *not* merely because of the media and other contributors to what Gramscians would call capitalist hegemony; not because of wider state ‘apparatuses’, as the Marxist ‘structuralists’ called the wider state, operating to thwart the interests of the masses. People did not see the radical alternative at that time as viable *because it was not*. Import controls and a semi‐planned economy (‘socialism in one country’) was not going to work. What Marxists see as the state screening out radical alternatives which are in the interest of the working‐class and oppressed may simply be the operation of democratic choice. In the UK, the Left in the early 1980s did a better job than the British state could ever do in discrediting radical ideas.

It is more sensible to put the very notion of the ‘capitalist state’ (as opposed to capitalist societies) into the dustbin of history and instead consider the biases induced by different state structures. There is no Marxist theory of *the* state which is (e.g.) capable of embracing both the UK state and the U.S. state. Instead, there are different types of state which ‐ as well as having the different consequences for policy explained in traditional perspectives on political institutions ‐ create biases over time in the production of policy which make it more or less difficult to achieve for example progressive health policy outcomes.

The point of the above vignette about Britain was to show the contrast with the U.S. Whether (or how much) the state can have autonomy from powerful social and economic interests is an *empirical* matter. In the UK, for example, parliament (which usually means the government with a majority in parliament) can be sovereign if it wants to be. It can pass a law in one day if it chooses (a recent example is the law to allow the government to take control of the one remaining steelworks in the UK, in Scunthorpe, when its Chinese owners were about to close it.) In the USA, by contrast, passing legislation is structurally (and culturally) more complex.

There, in a nutshell, is the main reason for differences in the UK and U.S. healthcare systems. Marxism adds nothing as an explanatory framework. It merely translates debates about power, structure and policy into pseudo‐Marxist language.

The U.S.'s decentralised institutions suggest that, on a superficial view, ‘pluralism’ reigns, with different interests having direct access. It would however be wrong to assume that the structure of the state allows these influences to be balanced out and moderated ‘fairly’: to depict the state as ‘referee’ is misleading. *The story of failed health reform in the U.S. is as good an illustration of this as any.*

## 9 Conclusion

The US state has traditionally offered entry points to conservative or ‘elite’ forces to obstruct progressive legislation. It can of course be pointed out that liberals also have entry points which may be of use in resisting when conservatives are trying to roll back the state. It is however liberal/left reformers who need a strong coordinating state. Resisting cuts incrementally is not the same as constructing a coherent welfare state.

The U.S. state illustrates how political structure and its mobilisation of bias creates a path‐dependency. In the U.S., this is functional for capitalism in that the wider state's pro‐capitalist bias (through multiple points of access for well‐financed powerful economic interests) is reinforced. But this is not because the U.S. is a ‘capitalist state’. It is because the U.S. state is too weak to challenge powerful corporate interests. That is the backdrop to the story of failed progressive health reform in the U.S. The U.S. state also has difficulty coordinating the ‘whole system’ when some of its parts are acting against that interest ‐ for example, when the behaviour of the health insurance industry in retarding rationalisation of the healthcare system is dysfunctional for *both* the economy *and* progressive health reform.

The U.S. political structure (decentralised and requiring a ‘concurrent majority’ to pass legislation) is the framework within which reformers are required to operate in order to maximise their chances of success, but that very framework thereby limits the nature and significance of ‘success’. A key example is the vicissitudes of Obama's health reform. Marmor [18] put it aptly, “The reforms that finally emerged from the Obama administration's initiative were the result of a year of nasty, demagogic and misleading claims in the US public forum, coupled with the complexities of crafting legislation that stood a chance of passing both the House of Representatives and the Senate. The resulting “hybrid” approach to healthcare reform produced a conservative strategy that ignores the experience of other wealthy democracies.”

Given this institutional dynamic and also the very American scepticism about unelected bureaucracy, periods of liberal reform tend to produce only weak bureaucracy running piecemeal social programmes. Creating a strong bureaucracy (of the sort required to run, e.g., a national health service) runs too much counter to the still‐dominant fear of the strong state which is independent of the separation of powers. Bureaucracy has to be directly beholden to particular political interests, meaning that (e.g.) it has to be re‐authorised and re‐appropriated regularly if not annually. This means that weak bureaucracy is easier to sweep away, and ‐ even if this does not happen ‐ political capital and energy is taken up defending limited gains rather than advancing them or making them into a coherent whole.

We are seeing yet another example of this phenomenon with the vitiation, if not the evisceration, of Obamacare. This may be only the beginning of a conservative attack upon equitable access to affordable healthcare [19].

## Ethics Statement

The author has nothing to report.

## Data Availability

The author has nothing to report.
